# Mitigating Effect of Ginger Extract on Survival Rate and Muscle Quality of Crucian Carp (*Carassius auratus*) Under Transportation Stress

**DOI:** 10.3390/ijms26167689

**Published:** 2025-08-08

**Authors:** Ling Peng, Chaoping Liu, Tao Yin, Shanbai Xiong, Juan You, Ru Liu, Qilin Huang

**Affiliations:** College of Food Science and Technology/National R&D Branch Center for Conventional Freshwater Fish Processing (Wuhan), Huazhong Agricultural University, Wuhan 430070, China; pengling@webmail.hzau.edu.cn (L.P.); liuchaoping2025@163.com (C.L.); xiongsb@mail.hzau.edu.cn (S.X.); juanyou@mail.hzau.edu.cn (J.Y.); liuru@mail.hzau.edu.cn (R.L.); hql@mail.hzau.edu.cn (Q.H.)

**Keywords:** muscle quality, oxidative stress, fish, individual packaging transportation

## Abstract

This study evaluated the effects of ginger extract, applied via four methods—direct addition, microencapsulation, and combinations with NaCl or eugenol—on stress responses and muscle quality in crucian carp during transportation. Among the treatments, microcapsules and the eugenol compound showed the best results, each achieving a 50% survival rate after 72 h. The microcapsule group provided prolonged antioxidant protection, stabilized water quality, reduced cortisol levels, suppressed pro-apoptotic gene expression (*hsp70*, *hsp90*, *il-6*, *caspase 3*, *caspase 8*, and *bax*), while upregulating the anti-apoptotic gene *bcl-2*. These alterations contributed to lower lactic acid accumulation and glycogen consumption, enhanced muscle shear force, reduced drip loss, and improved structural integrity of the gill, liver, and muscle tissues. The eugenol group effectively limited ammonia nitrogen accumulation, decreased glutathione peroxidase activity, and downregulated stress and apoptosis-related genes (*bax, caspase 3*, and *caspase 9*), resulting in reduced tissue damage. In contrast, the NaCl compound group accelerated water quality deterioration, increased TDS (total dissolved solids), lowered dissolved oxygen, and weakened stress resistance, leading to more severe tissue damage. Overall, microencapsulation or eugenol co-application were the most effective strategies for enhancing survival and maintaining muscle quality during transportation.

## 1. Introduction

The rapid expansion of e-commerce logistics has led to the growing adoption of individual live fish transportation as a preferred method for fresh food delivery [1]. By enabling direct, point-to-point distribution from production sites to consumers, this model increases economic efficiency for farmers while improving accessibility and time savings for buyers. However, this process inevitably exposes fish to a range of environmental stressors, such as heat [2], hypoxia [3], and ammonia nitrogen [4], which can trigger strong physiological stress responses, thereby leading to oxidative stress, metabolic disturbances, muscle quality deteriorate, and increased mortality rates post-transport [5,6].

To mitigate transport-induced stress, various strategies have been developed, including environmental optimization and the application of anti-stress agents [7,8,9]. NaCl addition is a widely used method that helps maintain osmotic balance, improving water quality (e.g., reducing ammonia toxicity) and suppressing pathogen proliferation (e.g., through ionic stress on microbes), thereby alleviated stress [9,10,11]. For instance, moderate salinity levels (2–4 g/L) have been shown to reduce cortisol and glucose levels and enhance antioxidant responses in freshwater species [12,13]. Another effective approach involves the use of natural anesthetics, such as eugenol, which have been proven to reduce oxidative damage and muscle quality deterioration in fish by slowing down metabolism during transport [9,14,15]. In recent years, plant-derived bioactive compounds have gained increasing attention for their potential in stress mitigation during fish transport [16,17]. Ginger (*Zingiber officinale*) extract, which is rich in bioactive compounds such as gingerols and curcuminoids, exhibits a wide range of biological activities, including antioxidant, anti-inflammatory, and neuroprotective effects [18,19,20]. It has also been widely used in fresh meat preservation as a natural antioxidant to scavenge free radicals and delay lipid oxidation [21]. These properties make it a promising candidate for alleviating transport-induced stress in aquaculture species. However, the application of ginger extract in live fish transport remains limited by challenges such as rapid degradation and short duration of action when administered directly into water.

Traditional direct addition methods have shown several limitations, such as short duration of action and the rapid oxidation or deactivation of active ingredients, which significantly reduce their practical application. Microcapsule embedding technology, which can delay the release of active ingredients and achieve long-term effects, has become a research hotspot [22]. Furthermore, Chen et al. [9] have been reported that different compound of anti-stress substances (NaCl, eugenol, and vitamin C (Vc)) can alleviate stress and improve muscle quality. Despite these advancements, systematic research on the various methods of ginger administration and their effects on fish survival rates and muscle quality remains limited.

This study aimed to evaluate the application efficacy of four methods of ginger exact—direct addition, microencapsulation, and combinations with NaCl or eugenol—in improving the survival rate, stress resistance, and muscle quality of crucian carp (Carassius auratus) during transportation of individual packaging. These findings are expected to contribute to the development of novel, natural, and practical strategies for mitigating transport-induced stress in live fish, thereby supporting sustainable aquaculture practices.

## 2. Results and Discussion

### 2.1. Free Radical Scavenging Ability of Ginger Treated with Microcapsules

**Figure 1:**
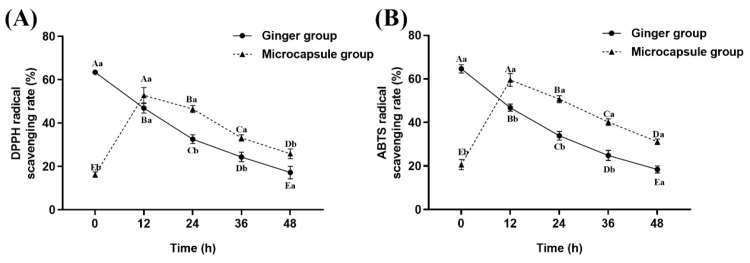
The radical scavenging rates of microcapsules on DPPH (**A**) and ABTS (**B**) at different time points. Notes: Different uppercase letters indicate significant differences within the group at different time points (*p* < 0.05), while different lowercase letters indicate significant differences between groups at the same time point (*p* < 0.05).

As illustrated in Figure 1, the antioxidant capacity of ginger extract progressively diminishes over time following its direct addition to water. This may be attributed to the fact that antioxidant compounds in ginger extract, such as gingerol and curcumin, are prone to hydrolysis and oxidation in aqueous solutions. These reactions can destroy active functional groups, thereby reduce antioxidant activity and weaken the ability to scavenge free radicals [23,24]. In the microcapsule group, the DPPH and ABTS radical scavenging activities of ginger extract initially increased and then decreased over time after being added to water. The microcapsule group exhibited the highest radical scavenging rate at 12 h after addition to the water. Moreover, its scavenging capacity remained significantly higher than that of the group treated with directly added ginger extract from 12 to 48 h. These findings suggest that microencapsulation enables a controlled release of active compounds from ginger extract in aqueous environments. As a result, the antioxidant activity is extended by preserving the availability of bioactive constituents over time.

### 2.2. Changes in Survival Rate of Fish During Transportation

**Figure 2:**
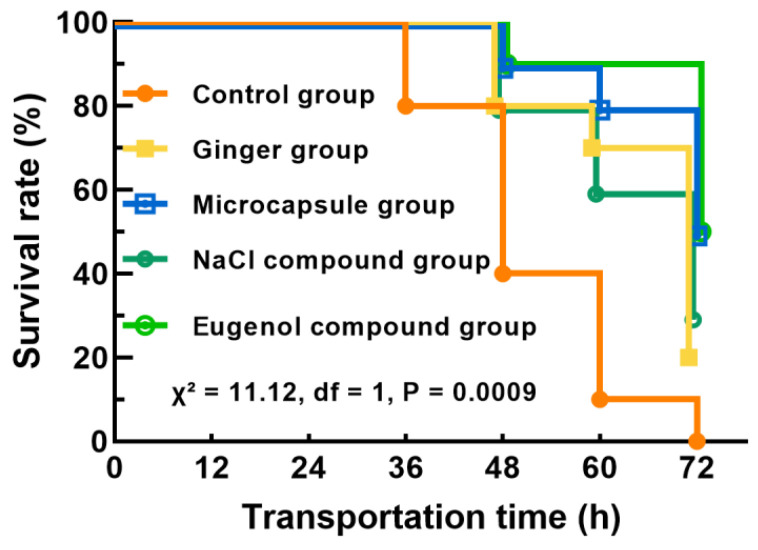
Changes in survival rate of fish during transportation.

As shown in Figure 2, the microcapsule and eugenol compound groups exhibited the highest survival rates at 72 h of transport, both reaching 50%. All groups except the control maintained 100% survival at 36 h. Survival rates at 60 h were notably elevated in all treatment groups compared to the control (10%), with the eugenol compound group reaching 90%, followed by the microcapsule (80%), ginger (70%), and NaCl compound (60%) groups. The addition of eugenol can reduce the metabolic activity rate in fish [9]. The antioxidant properties of ginger, especially when addition in microencapsulated form, may offer prolonged antioxidant effects (Figure 1). While NaCl can mitigate stress by enhancing osmoregulatory function [9], sustained exposure to elevated salinity levels may cause damage to gill tissues. Therefore, the eugenol compound and microcapsule group exhibited a higher survival rate compared to the other groups (Figure 2). After 72 h of transport, the survival rate of the control group dropped to 0, while the ginger extract group and the NaCl compound group-maintained survival rates of 20% and 30%, respectively. This suggests that either ginger extract alone or in combination with NaCl provides limited effective during prolonged transportation. In contrast, the microcapsule group and the eugenol compound group exhibited the highest survival rates, indicating that these two treatments offer superior anti-stress effects and can significantly improve the survival of fish during long-term transport.

### 2.3. Changes in Water Parameters During Transportation

#### 2.3.1. Dissolved Oxygen

**Figure 3:**
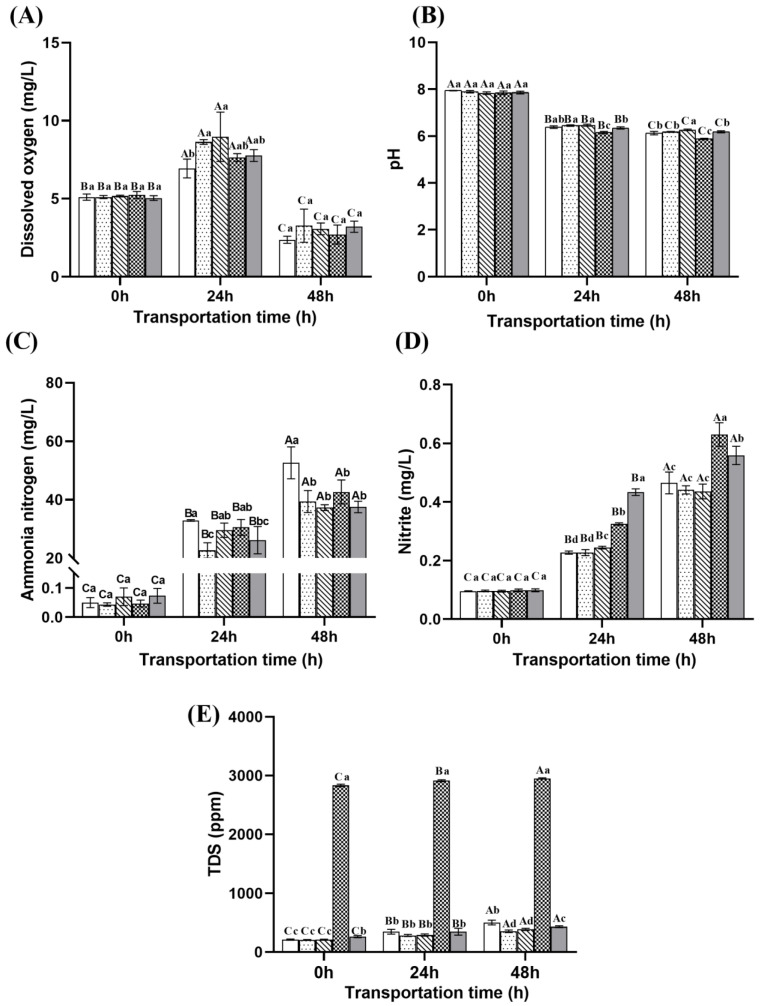
Changes in water quality parameters during transportation: (**A**) changes in dissolved oxygen during transportation; (**B**) changes in pH during transportation; (**C**) changes in ammonia nitrogen during transportation; (**D**) changes in nitrite during transportation; (**E**) changes in TDS during transportation. Note: Different uppercase letters indicate significant differences within the group at different time points (*p* < 0.05), while different lowercase letters indicate significant differences between groups at the same time point (*p* < 0.05). 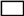: Control group; 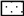: Ginger group; 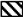: Microcapsule group; 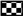: NaCl compound group; 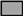: Eugenol compound group.

The dissolved oxygen level in the water exhibited an initial rise followed by a decline with prolonged transportation, as illustrated in Figure 3A. Notably, the highest dissolved oxygen concentration was observed at 24 h. This can be attributed to the early stage of transportation, during which the rate of oxygen dissolution into the water exceeded the total oxygen consumption by both fish and microorganisms. After 48 h of live transport, the concentrations in the ginger extract group, microcapsule group, NaCl compound group, and eugenol compound group were slightly higher than that of the control group, measuring 3.27, 3.07, 2.70, and 3.20 mg/L, respectively. However, these differences were not statistically significant (*p* > 0.05).

#### 2.3.2. pH

As shown in Figure 3B, the pH of the water gradually decreased over the course of transportation, shifting from slightly alkaline to slightly acidic. More precisely, after transportation for 24 h, the NaCl compound group exhibited the lowest pH value at 6.15, which was significantly lower than that of both the ginger extract group (6.47) and the microcapsule group (6.47) (*p* < 0.05). After 48 h of transportation, the microcapsule group maintained the highest pH value at 6.27. This is likely due to the continuous release of gingerol from the microcapsules, which inhibits lipid peroxidation and reduces the production of acidic metabolites such as malondialdehyde. In contrast, the pH in the NaCl compound group dropped to the lowest level at 5.88, indicating that osmotic regulation alone may exacerbate metabolic acidosis [25]. This may be attributed to the Cl^−^ ions in the NaCl compound group competing with HCO_3_^−^, thereby weakening the buffering capacity of bicarbonate [26]. Furthermore, the salinity stress may increase ammonia and urea excretion as part of disturbed nitrogen metabolism. The conversion of ammonia to ammonium and associated proton release contributes acid equivalents, further lowering pH in the water [27,28].

#### 2.3.3. Ammonia Nitrogen

**Figure 4:**
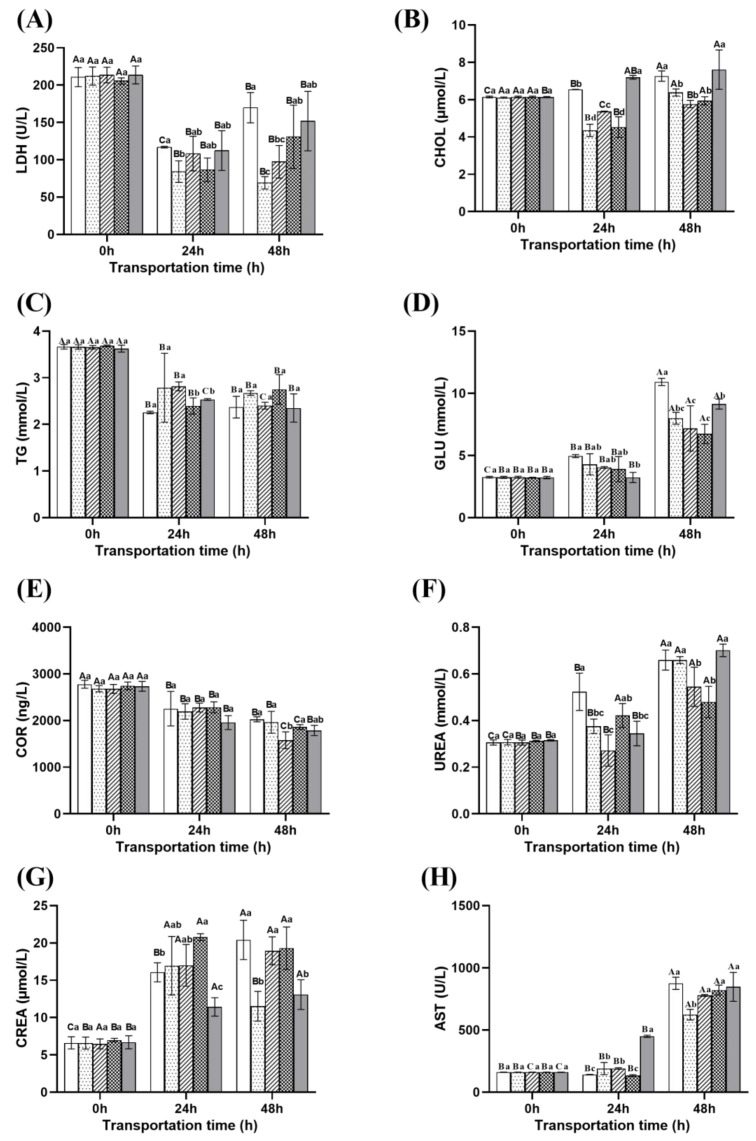
Changes in biochemical parameters of fish during different transportation. (**A**) changes in LDH content of fish during transportation; (**B**) changes in CHOL content of fish during transportation; (**C**) changes in TG content of fish during transportation; (**D**) changes in GLU content of fish during transportation; (**E**) changes in COR content of fish during transportation; (**F**) changes in UREA content of fish during transportation; (**G**) changes in CREA content of fish during transportation; (**H**) changes in AST content of fish during transportation. Note: Different uppercase letters indicate significant differences within the group at different time points (*p* < 0.05), while different lowercase letters indicate significant differences between groups at the same time point (*p* < 0.05). 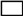: Control group; 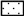: Ginger group; 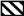: Microcapsule group; 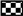: NaCl compound group; 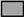: Eugenol compound group.

**Figure 5:**
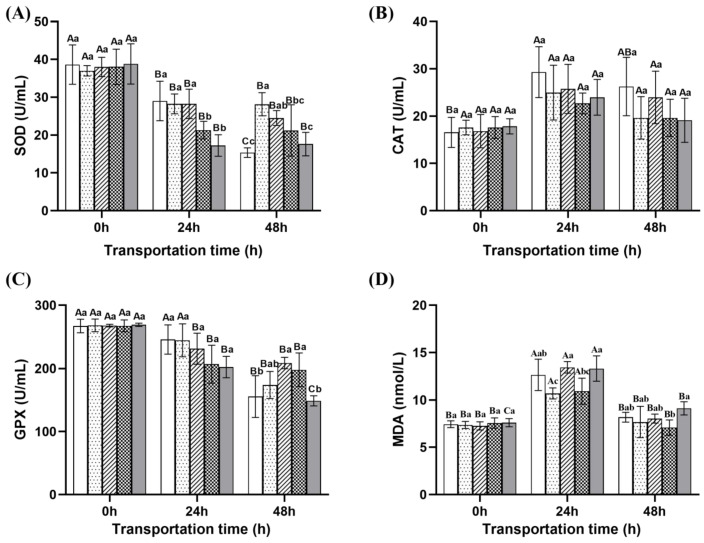
Changes in redox parameters of fish during different transportation. (**A**) Changes in SOD content of fish during transportation; (**B**) changes in CAT content of fish during transportation; (**C**) changes in GPX content of fish during transportation; (**D**) changes in MDA content of fish during transportation. Note: Different uppercase letters indicate significant differences within the group at different time points (*p* < 0.05), while different lowercase letters indicate significant differences between groups at the same time point (*p* < 0.05). 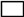: Control group; 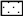: Ginger group; 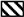: Microcapsule group; 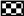: NaCl com pound group; 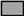: Eugenol compound group.

**Figure 6:**
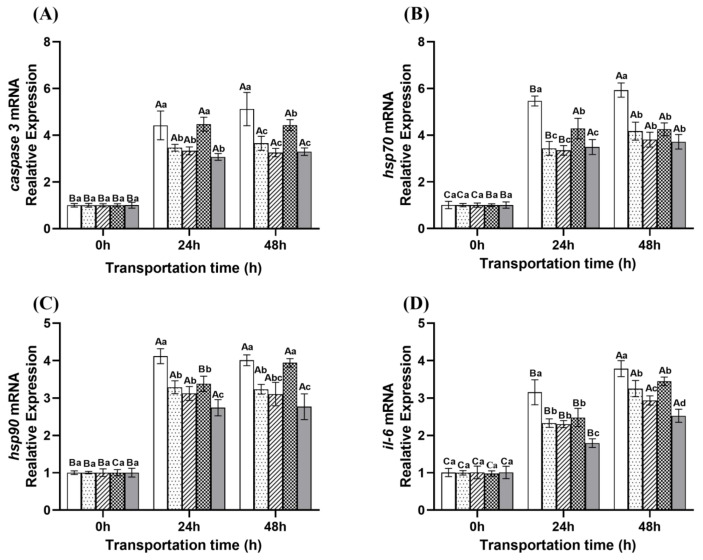
Changes in the gene expression levels of *caspase 3* (**A**), *hsp70* (**B**), *hsp90* (**C**), and *il-6* (**D**) in the blood of fish during transportation. Note: Different uppercase letters indicate significant differences within the group at different time points (*p* < 0.05), while different lowercase letters indicate significant differences between groups at the same time point (*p* < 0.05). 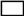: Control group; 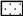: Ginger group; 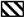: Microcapsule group; 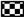: NaCl compound group; 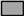: Eugenol compound group.

The concentration of ammonia nitrogen in the water showed an increasing trend with the extension of transportation time Figure 3C. The ammonia nitrogen concentration in the control group reached a peak of 52.65 mg/L, significantly higher than that of each treatment group (*p* < 0.05). Furthermore, the microcapsule group exhibited the lowest accumulation of ammonia nitrogen, with a concentration of 37.3 mg/L, suggesting that the microencapsulation technique is effective in suppressing ammonia nitrogen production. Notably, the ammonia nitrogen concentration in the NaCl compound group was 42.66 mg/L, which was lower than that of the control group but slightly higher than the other three treatment groups (ginger, microcapsule, and eugenol compound group). This may be attributed to the 3 g/L salinity increasing the energy demand for ion transport in fish, thereby accelerating protein catabolism to meet energy requirements and consequently leading to increased ammonia production [29]. The accumulation of ammonia nitrogen in the aquatic environment can exert toxic effects on fish by disrupting physiological homeostasis [30]. Elevated ammonia levels may impair gill function, damage liver and muscle tissue structures, and interfere with normal metabolic processes [30,31,32]. These physiological disturbances often trigger a cascade of stress responses, including increased cortisol secretion (Figure 4), oxidative stress (Figure 5), and altered immune function (Figure 6), ultimately compromising fish survival and muscle quality [33,34,35].

#### 2.3.4. Nitrite

As shown in Figure 3D, the nitrite concentration in the water shows an upward trend with the extension of transportation time. At 48 h, the nitrite concentrations in the NaCl compound group (0.63 mg/L) and eugenol compound group (0.56 mg/L) were significantly higher than those in the control group (0.47 mg/L), ginger group (0.44 mg/L), and microcapsule group (0.44 mg/L). The possible reason is that the addition of NaCl changes the salinity of the water and affects the activity of nitrifying bacteria, while eugenol, although having certain antibacterial and antioxidant effects, may not have a significant effect on promoting the nitrification process.

#### 2.3.5. TDS

As shown in Figure 3E, the TDS (total dissolved solids) levels in the water exhibited an increasing trend with the extension of transportation time. The addition of NaCl significantly increased salinity, resulting in a notable rise in TDS values. At the beginning of transportation (0 h), the TDS level in the NaCl compound group had already reached 2833.33 ppm, and further increased to 2950 ppm at 48 h. In contrast, after 48 h of transportation, the TDS levels in the ginger group, microcapsule group, and eugenol compound group were significantly lower than that of the control group (*p* < 0.05). TDS refers to total dissolved solids, which includes dissolved substances such as ammonia, ions, and small organic molecules that may be excreted by fish during stress [36]. Elevated TDS levels often reflect the accumulation of metabolic waste and can serve as an indirect indicator of deteriorating water quality, increased physiological stress, and impaired osmoregulatory function in fish [37,38]. The addition of NaCl significantly increased the ionic concentration, thereby leading to a marked rise in TDS. Moreover, the elevated concentration of ammonia nitrogen also contributed to the increase in TDS (Figure 3).

### 2.4. Changes in Blood Stress Indicators of Fish During Transportation

#### 2.4.1. Biochemical Indicators

(1) Energy metabolism parameters

As shown in Figure 4A, the LDH (lactate dehydrogenase) concentrations in the control, NaCl compound, and eugenol compound groups initially decreased and then increased as transportation time was extended. The LDH concentration of ginger group and microcapsule group showed a decreasing trend with the prolongation of transportation time. At 48 h of transportation, the LDH concentration in the control group was the highest, while the LDH concentrations in the NaCl and eugenol compound groups were significantly higher than those in the ginger and microcapsule groups (*p* < 0.05). The possible reason is that the compound group of eugenol reduces metabolic rate in the early stage, but the effect weakens in the later stage, leading to stress rebound. The NaCl compound group may experience increased metabolic pressure in the later stage due to the additional burden of ion regulation, leading to a decrease in membrane stability and an increase in LDH.

The CHOL (cholesterol) concentration of the control group and the eugenol compound group (Figure 4B) showed an increasing trend with the prolongation of transportation time. The ginger group and NaCl compound group showed an initial decrease followed by a increase trend. The TG concentration (Figure 4C) showed a decreasing trend with the prolongation of transportation time. The concentration of GLU (Figure 4D) shows an upward trend with the prolongation of transportation time. After transportation for 48 h, the CHOL and GLU concentrations in the control group and eugenol compound group were significantly higher than those in the ginger group, microcapsule group, and NaCl compound group (*p* < 0.05), and the TG (triglyceride) concentration was lower than that in the ginger group, microcapsule group, and NaCl compound group. This may be due to the anesthetic effect of the compound group of eugenol, which reduces the efficiency of glycolysis energy supply, forcing the liver to accelerate fatty acid β-oxidation compensation, generate excess acetyl CoA, activate the HMG CoA reductase pathway, thereby accelerating TG decomposition and promoting cholesterol synthesis [39]. At the same time, its anesthetic effect inhibits insulin secretion, leading to impaired glucose utilization and resulting in blood glucose accumulation [40].

(2) Hormone parameter

From Figure 4E, it can be seen that there is a decrease in serum COR (cortisol) concentration during transportation. Furthermore, the content of COR was lowest in microcapsule group compared with other groups after transportation for 48 h. This may be attributed to gingerol inhibiting the NF-κB inflammatory pathway [17], resulting in a 22.2% decrease in COR production compared to the control group. The anesthetic effect of the compound group of eugenol has been attenuated at 48 h, but the early metabolic inhibition reduces the stress accumulation effect and lowers COR secretion; Although the osmotic regulation of the NaCl compound group can temporarily alleviate ion imbalance, its metabolic cost partially offsets the anti-stress effect through energy competition, oxidative damage, and other pathways, and the COR inhibitory effect is weak [41].

(3) Renal metabolism parameters

As shown in Figure 4F,G, the concentrations of UREA and CREA (creatinine) show an upward trend with the prolongation of transportation time. At 48 h, the concentration of UREA in the control group, ginger group, and eugenol compound group was significantly higher than that in the microcapsule group and NaCl compound group (*p* < 0.05); The CREA concentration in the control group and NaCl compound group was significantly higher than that in the ginger group and eugenol compound group (*p* < 0.05). This phenomenon indicates that directly added ginger extract cannot sustainably inhibit protein hydrolysis due to rapid degradation, while the microcapsule group can significantly inhibit protein degradation and maintain kidney function through long-term antioxidant and anti-inflammatory effects. The eugenol compound group can effectively reduce creatinine production by reducing muscle activity, but long-term metabolic inhibition may lead to negative energy balance, forcing fish to accelerate protein breakdown in the later stage. The NaCl compound group can stabilize the ion transport function of gills, reduce stress-induced protein degradation metabolism, and thus reduce the generation of urea precursors [42]; However, long-term exposure to high salinity can induce a decrease in glomerular filtration rate, leading to the accumulation of CREA.

(4) Liver metabolism parameters

As shown in Figure 4H, the concentration of AST (aspartate aminotransferase) increases with the prolongation of transport time, reflecting an increase in membrane permeability of liver cells. At 48 h, the AST concentration in the control group reached 875.65 U/L, while the ginger group (623.8 U/L) showed the best performance in the treatment group, followed by the Microcapsule group (778.28 U/L), NaCl compound group (821.32 U/L), and eugenol compound group (847.14 U/L). AST accumulation is a key marker of transport induced liver injury, and ginger extract achieves efficient protection through multi-target anti-inflammatory antioxidant mechanisms. Osmotic regulation and anesthesia strategies may exacerbate liver load due to metabolic compensatory effects [43].

#### 2.4.2. Redox Parameters

As shown in Figure 5, the activities of SOD (superoxide dismutase) and GPX (glutathione peroxidase) showed a decreasing trend with the prolongation of transport time; After 48 h of transportation, the SOD and GPX activities in the control group were significantly lower than those in the microcapsule group and NaCl compound group (*p* < 0.05). The activities of SOD and GPX in the compound group of eugenol were higher than those in the control group, but lower than the other three groups. The activity of CAT (catalase) and the content of MDA (malondialdehyde) showed a trend of first increasing and then decreasing with the prolongation of transportation time. After 48 h of transportation, the CAT activity of the control group was the highest, and there was no significant difference between the groups (*p* > 0.05). The MDA content in the compound group of eugenol was higher than the other four groups.

The SOD and GPX activities were higher in the ginger group, microcapsule group, and NaCl compound group, indicating that ginger extract can improve the antioxidant capacity of fish and effectively cope with environmental stress. The NaCl compound group may indirectly reduce GPX consumption by stabilizing gill ion transport function, reducing ROS bursts caused by mitochondrial calcium overload [44]. The MDA content in the compound group of eugenol is higher, possibly due to the anesthetic effect of eugenol, which inhibits the activity of mitochondrial respiratory chain complex I, increases electron leakage [45], and inhibits GSH synthesis, leading to impaired GPX function and weakened H_2_O_2_ clearance ability, forming a vicious cycle of free radical accumulation membrane damage [46].

#### 2.4.3. Gene Expression

As shown in Figure 6, the transcription levels of *hsp70* and *hsp90* showed an upward trend with prolonged transport time. The relative expression levels of *hsp70* and *hsp90* in the control group were significantly higher than those in the treatment group at 24 and 48 h (*p* < 0.05). This indicates that the stress response of the control group is most intense during transportation, and cells need to synthesize large amounts of HSP70 and HSP90 to maintain cell stability and function. In contrast, the expression levels of *hsp70* and *hsp90* were lower in the ginger group, microcapsule group, and eugenol compound group, indicating that these treatment methods can effectively alleviate oxidative stress in fish during transportation. Although the NaCl compound group showed slightly higher *hsp90* expression than other treatment groups, the overall stress level was still lower than that of the control group. The transcription levels of *caspase 3* and *il-6* showed an upward trend with prolonged transport time. The relative expression levels of *caspase 3* and *il-6* in the control group were significantly higher than those in the treatment group at 24 and 48 h (*p* < 0.05), indicating that the apoptosis and inflammatory response in the control group were more severe. At 48 h, the relative expression levels of *caspase 3* were the lowest in the microcapsule group and the eugenol compound group, indicating that the microcapsule group and the eugenol compound group can more effectively inhibit cell apoptosis [47]. The lowest expression level of *il-6* was observed in the compound group of eugenol, indicating that the compound group of eugenol has a more significant anti-inflammatory effect [48]. The high levels of *caspase 3* and *il-6* in the NaCl compound group indicate that the use of NaCl to some extent exacerbates cellular stress and dehydration, increasing inflammatory response and cell apoptosis.

### 2.5. Histological Changes in Gill and Liver of Fish After 48 h of Transportation

**Figure 7:**
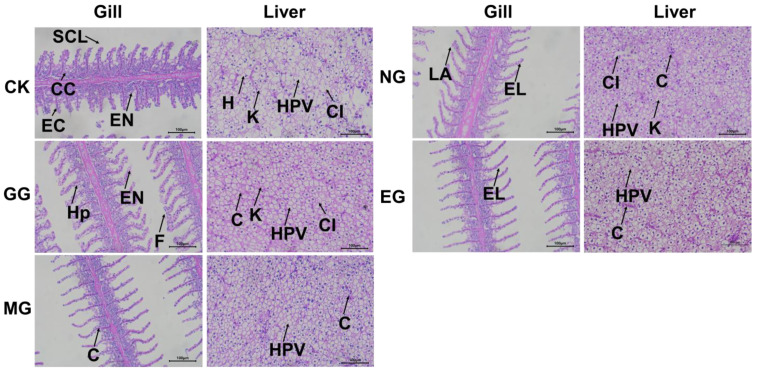
Changes in the histological structure of the gill and liver of fish after 48 h of transport. CK: Control group; GG: Ginger group; MG: Microcapsule group; NG: NaCl compound group; EG: Eugenol compound group. SCL: secondary lamellae; CC: chloride cells; EC: epithelial cell; EN: epithelial necrosis; EL: epithelial lifting; H: hepatocyte; K: karyolysis; Hp: hyperplasia; F: fusion of the secondary lamellae; HPV: hepatocellular vacuolation; C: congestion; LA: lamellar aneurysm; CI: indistinct cellular outline.

As shown in Figure 7, after 48 h of transportation, the gill filaments in the control group were severely damaged and swollen and ruptured. The liver tissue exhibited extensive cellular damage, with large, continuous vacuolated regions and loosely arranged, disordered hepatocytes. All treatment groups alleviated gill and liver damage to varying degrees. Among them, the microcapsule group and the eugenol compound group showed more pronounced protective effects. In these two groups, the gill filaments were neatly arranged, with only mild bending observed in a few filaments. Hepatocytes maintained relatively intact structures with clearer nuclei. In contrast, the ginger group and the NaCl compound group showed moderate damage. Most gill filaments were bent but still relatively well-aligned. Dilated blood vessels, swollen mucus cells, and more severe chloride cell proliferation and vacuolization were observed compared to the microcapsule group. In the liver, a small number of vacuoles were present, with more prominent nuclear shrinkage and blurred cellular boundaries. The gill and liver tissues in the microcapsule group and the eugenol compound group exhibited the most intact and well-defined structures, indicating a more effective alleviation of oxidative stress and enhanced protection of tissue integrity. The more severe structural damage observed in the gill and liver tissues of the ginger extract group and the NaCl compound group may be attributed to different underlying factors. In the ginger group, the direct addition of ginger extract to the water likely led to its rapid degradation, resulting in weakened antioxidant protection during the later stages of transportation (>24 h) [23,24]. This decline may have contributed to a rebound in the inflammatory cytokine IL-6 levels, causing local blood sinus dilation and widened intercellular spaces in gill epithelial cells. In the NaCl compound group, prolonged exposure to a high-salinity environment may have increased the osmotic burden on gill and liver tissues, ultimately exacerbating structural damage [41].

### 2.6. Changes in Muscle Quality of Fish During Transportation

#### 2.6.1. Cellular Structure

**Figure 8:**
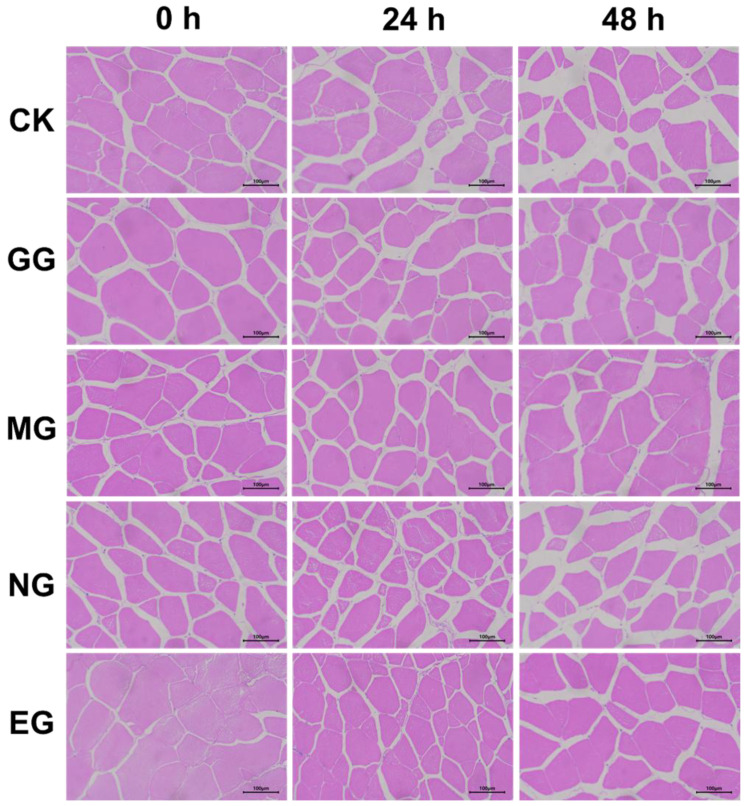
Changes in the muscle cellular structure of fish during transportation. CK: Control group; GG: Ginger group; MG: Microcapsule group; NG: NaCl compound group; EG: Eugenol compound group.

The control group and the NaCl compound group exhibited similar results, both showing noticeable muscle cell fragmentation and enlarged intercellular spaces (Figure 8, Table S1). In comparison, the ginger group displayed slightly lower degrees of cell fragmentation and smaller intercellular spaces than those observed in the control and NaCl compound groups. The microcapsule group and the eugenol compound group demonstrated relatively smaller intercellular spaces and more compact muscle fiber arrangement, although the gaps in the microcapsule group were slightly larger than those in the eugenol compound group. Among all groups, the eugenol compound group exhibited the most effective preservation of muscle cell structure. This may be due to the anesthetic effect of eugenol, which reduces the frequency of convulsive muscle contractions, thereby minimizing mechanical damage to muscle fibers. In addition, the synergistic action of ginger extract helps mitigate oxidative stress and inhibits the NF-κB mediated release of inflammatory cytokines, ultimately alleviating the damage of muscle [49].

#### 2.6.2. Physical Properties

(1) Shear force and drip loss

**Figure 9:**
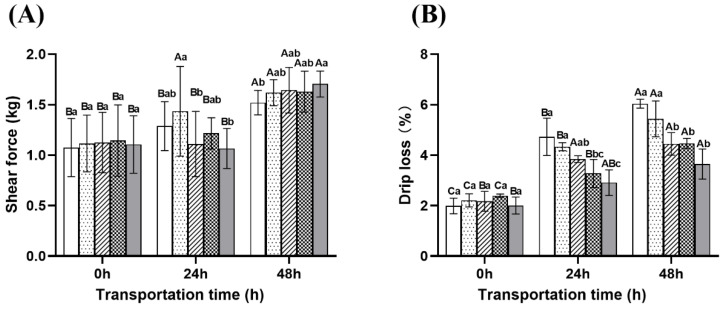
Changes in the shear force (**A**) and drip loss (**B**) of fish muscle during transportation. Note: Different uppercase letters indicate significant differences within the group at different time points (*p* < 0.05), while different lowercase letters indicate significant differences between groups at the same time point (*p* < 0.05). 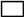: Control group; 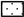: Ginger group; 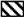: Microcapsule group; 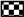: NaCl compound group; 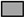: Eugenol compound group.

At 48 h, the shear force of the treatment group was significantly higher than that of the control group (1.52 kg), with the eugenol compound group having the highest shear force (1.71 kg), while the ginger group, microcapsule group, and NaCl compound group had shear forces of 1.62 kg, 1.64 kg, and 1.63 kg, respectively (Figure 9A). This may be attributed to the anesthetic effects significantly reducing fish activity levels, thereby minimizing damage to muscle fibers [50]. Additionally, the suppression of glycolysis may have delayed the degradation of myofibrillar proteins, helping to preserve muscle structural integrity [50]. This indicates that the combination of eugenol fish ginger extract may further optimize the anti-stress effect to a certain extent.

As shown in Figure 9B, the drip loss of muscle increased with the extension of transportation time. After 48 h of transportation, the eugenol compound group exhibited the lowest drip loss (3.65%), followed by the microcapsule group (4.45%), NaCl combination group (4.46%), ginger extract group (5.44%), and the control group (6.04%). The superior performance of the eugenol compound group may be attributed to its anesthetic effect, which reduces fish activity, thereby lowering energy metabolism and lactic acid accumulation [9]. The effectiveness of the microcapsule group might be due to the continuous release of active compounds, maintaining a stable antioxidant effect and reducing oxidative damage to muscle cell membranes (Figure 1). The NaCl compound group likely maintained cellular structure by regulating osmotic pressure [51], while the ginger extract group showed less effectiveness in the later stages, possibly due to the rapid degradation of its antioxidant compounds (Figure 1) [18].

(2) Color

As shown in Table 1, both the W value and L* value of fish muscle increased with extended transportation time. After 48 h of transportation, the eugenol compound group showed the highest muscle L*, with an L* value of 52.37, which was significantly higher than that of the control group (50.94). Furthermore, with the extension of transportation time from 24 to 48 h, the a* value increased in the control group, whereas a decrease was observed in the treatment groups. This effect may be attributed to the addition of ginger extract, which helps alleviate stress (Figure 4).

#### 2.6.3. Muscle Glycogen, pH, and Lactate

**Figure 10:**
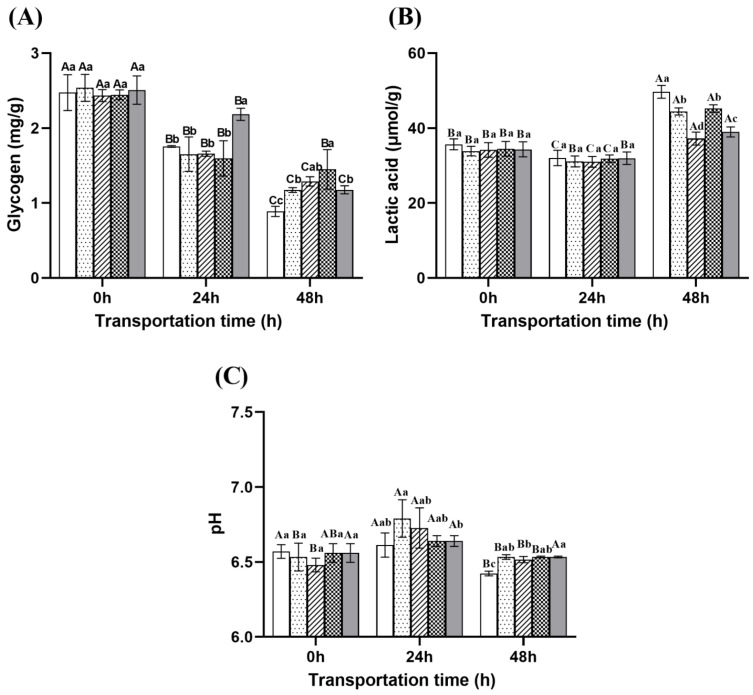
Changes in muscle glycogen (**A**), lactic acid (**B**) and pH (**C**) in the dorsal muscle during transportation. Note: Different uppercase letters indicate significant differences within the group at different time points (*p* < 0.05), while different lowercase letters indicate significant differences between groups at the same time point (*p* < 0.05). 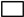: Control group; 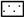: Ginger group; 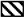: Microcapsule group; 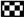: NaCl compound group; 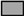: Eugenol compound group.

As shown in Figure 10A, muscle glycogen content gradually decreased with the prolongation of transportation time. At 24 h, the eugenol compound group exhibited significantly higher glycogen levels compared to the other groups. However, by 48 h, its glycogen content dropped sharply to 1.18 mg/g, which was significantly lower than that of the NaCl compound group (1.45 mg/g). A significant increase in lactate content was observed as the transportation time extended from 0 to 48 h, with the control group exhibiting the highest accumulation, reaching 51.49 μmol/g (Figure 10B). Correspondingly, the pH level in fish muscle decreased significantly (Figure 10C). These results indicate that the microcapsule group and the eugenol compound group were most effective in controlling lactate accumulation during transportation, thereby alleviating stress responses and helping to maintain acid–base balance in the muscle.

#### 2.6.4. ATP and Its Metabolites

According to Table 2, the ATP content shows a trend of first increasing and then decreasing with the extension of transport time, while the ADP content continues to decrease. At 48 h, the ATP content of the eugenol compound group (5.58 mg/100 g) was significantly higher than that of the other groups, which may be due to its anesthetic effect, reducing energy consumption and inhibiting ATPase activity, delaying ATP degradation [52]. The content of IMP, an umami substance, continues to increase in each group; The AMP content in the control group first increased and then decreased, while the AMP content in the ginger group, microcapsule group, and NaCl compound group continued to decrease. The AMP content in the eugenol compound group first decreased and then increased, reaching a maximum value of 6.13 mg/100 g at 48 h, significantly higher than other groups (*p* < 0.05). This may be due to the release of metabolic inhibition in the late stage of anesthesia, insufficient ATP synthesis triggering excessive activation of the AMP kinase (AMPK) pathway, and feedback increasing AMP production [53]. Moreover, the content of Hx and HxR shows an upward trend with the extension of transportation time. Compared with the control group, the treatment group significantly inhibited the production of bitter substances Hx and HxR (*p* < 0.05). From the perspective of K value alone, at 24 h, the eugenol compound group had a significant effect on maintaining muscle freshness and reducing K value (*p* < 0.05). At 48 h, there was no significant difference in K value among the groups (*p* < 0.05), suggesting that ginger extract, NaCl, and eugenol exert effective stress-relieving effects during the early stage of transportation. However, with prolonged transportation, the freshness of fish muscle may be gradually compromised. This deterioration can be attributed to several factors, including the degradation of active components in ginger extract, the increased physiological burden caused by sustained high salinity, and the metabolic compensation induced by eugenol anesthesia.

### 2.7. Changes in Apoptosis of Fish During Transportation

**Figure 11:**
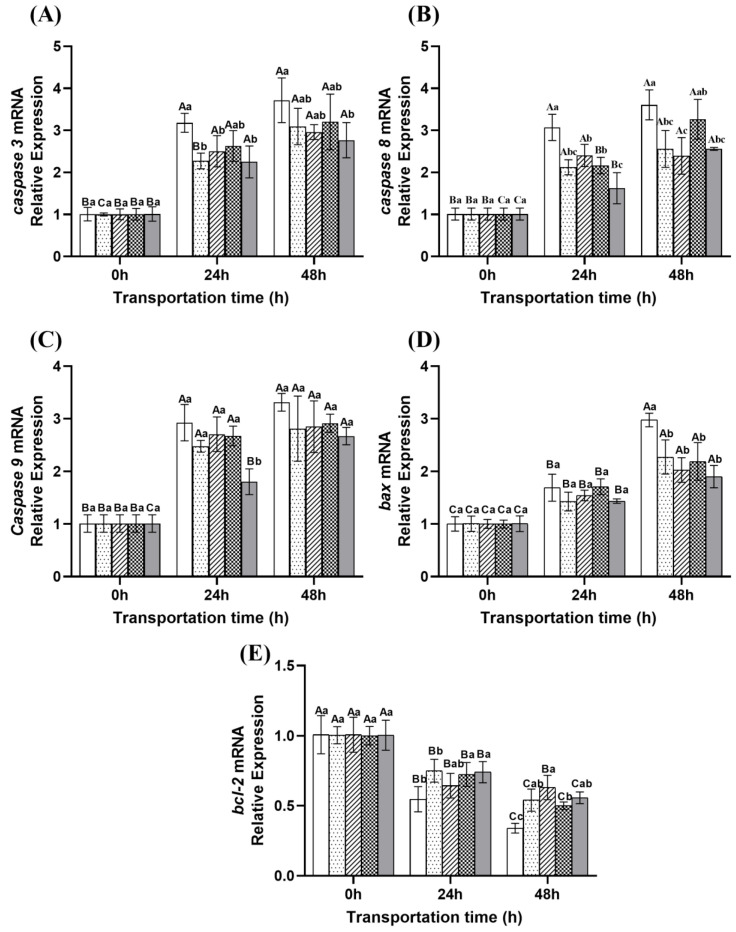
Changes in the expression levels of *caspase 3* (**A**), *caspase 8* (**B**), *caspase 9* (**C**), *bax* (**D**), and *bcl-2* (**E**) in the dorsal muscle during transportation. Note: Different uppercase letters indicate significant differences within the group at different time points (*p* < 0.05), while different lowercase letters indicate significant differences between groups at the same time point (*p* < 0.05). 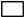: Control group; 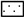: Ginger group; 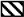: Microcapsule group; 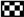: NaCl compound group; 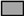: Eugenol compound group.

The expression patterns of apoptosis-related genes during transportation are shown in Figure 11. The transcription levels of pro-apoptotic genes *caspase 3*, *caspase 8*, *caspase 9* and *bax* showed an increasing trend with prolonged transportation time, whereas the transcription level of the anti-apoptotic gene *bcl-2* gradually decreased over time. These results indicate that transportation induces the activation of apoptotic pathways in fish, likely as a physiological response to prolonged stress [4,54]. At 24 h, the ginger group and eugenol compound group exhibited the lowest relative expression levels of *caspase 3* (*p* < 0.05), indicating superior anti-stress effects during the early stage of transportation (0–24 h) compared to the microcapsule and NaCl compound groups. At 48 h, the microcapsule group showed the lowest *caspase 8* expression level (*p* < 0.05), followed by the eugenol compound group. Additionally, the eugenol compound group had the lowest relative expression levels of *caspase 3* and *caspase 9* at this time point, with the sustained-release group ranking second. These results indicate that the eugenol compound group and the microcapsule group exhibited a more pronounced inhibitory effect on apoptosis, whereas the NaCl compound group and the ginger group were relatively less effective. The enhanced anti-apoptotic effect observed in the eugenol compound group may be attributed to the combined action of anesthetic properties and the mitochondrial protective effects of ginger extract, forming a dual regulatory barrier that blocks apoptotic signal transduction [55]. Furthermore, Ginger extract can exert continuous antioxidant protection and modulate apoptosis by activating the Nrf2 signaling pathway, thereby downregulating the pro-apoptotic gene *bax* and upregulating the anti-apoptotic gene *bcl-2*. This mechanism effectively reduces oxidative stress, inflammation, and apoptosis [49]. The microcapsule group effectively prolonged the antioxidant activity of ginger extract by scavenging superoxide anions (O_2_^−^), thereby preventing the decline of mitochondrial membrane potential and inhibiting the release of cytochrome c, thereby decreased the cellular apoptosis [56].

## 3. Materials and Methods

### 3.1. Materials

The crucian carp used in this experiment were all purchased from the Wuhan Smart Agricultural Research Aquatic Products Base during October 2024 and January 2025. The average weight of the crucian carp was (220 ± 30) g, and the body length was (23 ± 3) cm.

The ginger extract used in this study was commercially purchased from Xian ReaHerb Bio-Tech Co., Ltd. (Xian, China). According to the product information provided by the manufacturer, it is an aqueous extract derived from *Zingiber officinale* rhizomes and contains 1% 6-gingerol (CAS No.: 23513-14-6) as the major bioactive compound.

### 3.2. Ethic Statement

All animal standard operating procedures have been approved by the Animal Ethics and Use Committee of Huazhong Agricultural University (HZAUF-2024-0042) and executed in accordance with the “Huazhong Agricultural University Laboratory Animal Care and Utilization Guidelines”.

### 3.3. Preparation of Microcapsules

The preparation of microcapsules was based on the method described by Zhang [57] with slight modifications. More precisely, two grams of sodium alginate were weighed and dissolved in 100 g of distilled water. The mixture was stirred thoroughly and then subjected to magnetic stirring in a constant-temperature water bath at 60 °C for 45 min, allowing the sodium alginate to fully hydrate and swell. Subsequently, carboxymethyl cellulose powder (1.5 g) was added, and the solution was further stirred under the same conditions until fully dissolved. The ginger extract was then added to the wall material solution at a core-to-wall ratio of 1:3, followed by manual stirring with a glass rod. The mixture was stirred evenly with a glass rod and ultrasonicated at 120 W and 25 °C for 10 min. Separately, calcium chloride (3 g) was dissolved in 100 mL of water. From a height of approximately 10 cm above the liquid surface, the core-wall mixture was dropped into the calcium chloride solution using a syringe at a rate of 1 mL/min. The solution was magnetically stirred at 45 °C for 20 min to allow microcapsule formation. The microcapsules were collected by filtration and freeze-dried for 48 h.

### 3.4. Determination of Free Radical Scavenging Ability

The DPPH and ABTS free radical scavenging ability of 0.1 mg/mL microcapsules (equivalent to 0.1 mg/mL ginger extract) were measured at different time points (0, 12, 24, 36, 48 h). The determination of free radical scavenging ability is referenced in [58,59].

### 3.5. Sample Preparation

Our previous unpublished results indicated that a concentration of 0.1 mg/mL ginger extract was most effective in reducing oxidative stress and preserving muscle quality. Accordingly, this concentration was adopted in the current study.

(1) Simulated Transportation Program

At the end of the fasting period, fish of similar size and a healthy condition were selected for the simulated transport experiment. The fish-to-water ratio was maintained at 1:3, using aerated tap water as the water source. The packaging procedure was as follows: prior to sealing, each transport bag was filled with aerated tap water or a sample solution of a predetermined concentration prepared using aerated tap water. The packaging specification is 1 tail/bag, exhaust the air in the fish bag, seal it with a thermoplastic sealing machine, and then fill the fish bag with oxygen to make the initial pressure in the fish bag reach about 12 kPa. Then put it into a foam incubator (290 × 170 × 190 mm), add an ice bag of 50% of the total weight of fish and water, and place it on a simulated transport platform for simulated transport.

(2) The Effects of Different Addition Methods of Ginger Extract on Survival Rate

Simulate transportation time up to 72 h, and calculate survival rate every 12 h. The specific treatment is as follows. Control group: inject aerated tap water without any other treatment. Ginger group: inject aerated tap water and add ginger extract (0.1 mg/mL). Microcapsule group: inject aerated tap water and add ginger extract microcapsules (0.1 mg/mL). NaCl compound group: inject aerated tap water, add ginger extract (0.1 mg/mL) and sodium chloride (3 g/L) [13]. Eugenol compound group: inject aerated tap water and add ginger extract (0.1 mg/mL) and eugenol (6 mg/L) [50]. A limitation of this study is that it only investigated the combined effects of NaCl or eugenol with ginger extract, without including treatment groups with NaCl or eugenol alone.

(3) The Effects of Different Addition Methods of Ginger Extract on Stress Response and Muscle Quality of Fish

Randomly select 75 fish and divide them into 5 groups of 15 fish each. Pack each fish in a fish bag. They are divided into a control group, ginger group, microcapsule group group, NaCl compound group, and eugenol compound group. Fish were randomly sampled from each group at 0, 24, and 48 h. Euthanasia was performed using 120 mg/L of MS-222 anesthetic. Blood was collected from the caudal vein using a 5 mL sterile syringe. Subsequently, the dorsal muscle was obtained after filleting the fish and used for further analysis. Furthermore, water quality parameters, blood stress parameters and muscle quality were taken at fixed intervals of 24 h to detect changes in water indicators, physiological and biochemical indicators, and muscle quality.

### 3.6. Survival Rate

After complete loss of breath, crucian carp can be considered dead [60], characterized by floating heads, overturned bellies, and other features. When conducting statistics, each group of crucian carp will be kept alive under corresponding experimental conditions. The statistical time points are at 0, 12, 24, 36, 48, 60, and 72 h after the start of preservation, and the number of fish in each group still alive at each time point will be counted. The formula for calculating survival rate is

$$w_{t}=\frac{n_{t}}{n}\times 100\%$$

*w_t_*: Survival rate of each group at different times;
*n_t_*: Survival quantity of each group at different times;
*n*: The initial quantity for each group is 10 in this experiment.

### 3.7. Determination of Water Indicators

Ammonia nitrogen concentration was measured using Nessler’s Reagent Method by ammonia nitrogen detector (YC7200-N, Shenzhen, China); pH was measured with Sigma pH meter (PH818, Dongguan, China); dissolved oxygen was measured with dissolved oxygen meter (AR8210, Dongguan, China). The nitrite concentration was determined using the N-(1-naphthyl) ethylenediamine dihydrochloride method by microplate reader (Multiskan SkyHigh, Thermo Fisher Scientific, Waltham, MA, USA).

### 3.8. Determination of Biochemical Parameter

The cortisol (COR) in serum was performed using the Shanghai Jingkang enzyme-linked immunosorbent assay kit, and the experiment was carried out according to the kit instructions. Glucose (GLU), cholesterol (CHOL), triglyceride (TG), aspartate aminotransferase (AST), lactic dehydrogenase (LDH), UREA, and creatinine (CREA) in serum were determined by fully automated biochemical analyzer (Chemray 240, Shenzhen Leidu Life Science Co., Ltd., Shenzhen, China).

### 3.9. Determination of Redox System Indicators

The activities of superoxide dismutase (SOD) and glutathione peroxidase (GPX), as well as the content of malondialdehyde (MDA), were determined using commercial assay kits provided by Beijing Solarbio Science & Technology Co., Ltd. (Beijing, China).

### 3.10. Observation of Tissue Structure

Histological examinations of the gill, liver, and muscle were conducted using HE staining and observed under a light microscope (Eclipse Ci, Nikon, Tokyo, Japan), following the methods of Peng et al. [61] and Zhong et al. [62]. Notably, gill tissue underwent decalcification in EDTA, during which the solution was replaced every three days until the tissues were fully softened.

### 3.11. Determination of mRNA Relative Expression of hsp70, hsp90, caspase3, and il-6 in Blood

#### 3.11.1. Total RNA Extraction

Whole blood was centrifuged and the supernatant discarded. Red blood cell lysis buffer was added, and the sample was thoroughly mixed and centrifuged again. This process was repeated 1–2 times until the supernatant became clear, and the final pellet was collected. Then, total RNA was extracted from the pellet using RNA extraction solution (Wuhan Servicebio technology Co., Ltd, Wuhan, China). Moreover, RNA concentration and purity were determined using a NanoDrop 2000 spectrophotometer (Thermo, Waltham, MA, USA).

#### 3.11.2. Reverse Transcription and Quantitative PCR

Reverse transcription was performed using 10 μL total RNA as template in a 20 μL reaction system containing 4 μL 5× SweScript All-in-One SuperMix for qPCR (G3329, Wuhan Servicebio technology Co., Ltd., Wuhan, China), 1 μL gDNA remover, and 5 μL RNase-free water. The reaction conditions were: 25 °C for 5 min, 42 °C for 30 min, and 85 °C for 5 s using a standard thermal cycler.

For qPCR, primers were prepared as 100 μM stock solutions and diluted to 10 μM working concentrations. Details of the primer sequences are listed in Table 3. The cDNA was diluted 3–5-fold with RNase-free water before use. The 20 μL qPCR reaction mixture contained 10 μL 2× qPCR premix (G3326, Wuhan Saiweier Biotechnology Co., Ltd., Wuhan, China), 0.4 μL forward primer, 0.4 μL reverse primer, 2.0 μL cDNA, and 7.2 μL ultrapure water. The cycling protocol was as follows: 95 °C for 30 s (initial denaturation), followed by 40 cycles of 95 °C for 15 s and 60 °C for 30 s (annealing/extension).

### 3.12. Determination Muscle Quality of Fish

(1) Shear Force

Shear force was measured following the method of Peng et al. [61], with slight modifications. A physical property analyzer equipped with an HDP-BS blade was used, with the testing speed set to 1 mm/s and the compression ratio fixed at 100%. The “Hold” mode was enabled to record the maximum force (kg) during the measurement.

(2) Drip Loss

Drip loss was determined using the hanging and weighing method. After sampling, the dorsal muscle was cut into uniform cubes (20 mm × 20 mm × 10 mm) and weighed. The fresh muscle samples were then suspended in a refrigerator at 4 °C. Muscle weights were recorded at 0 and 24 h. Drip loss was calculated using the following formula:

$$Driploss(\%)=\frac{M1-M2}{M1}\times 100\%$$

where M1 is the starting mass, and M2 is the ending mass

(3) Color

Muscle samples from each group of fish were cut into uniform pieces (20 mm × 20 mm × 10 mm). A colorimeter was calibrated using a standard white plate prior to measurement. The L* (lightness), a* (red or green), and b* (yellow or blue) values were recorded. The whiteness value (*W*) was calculated using the following formula:

$$W=100-\sqrt{{\left(100-L^{*}\right)}^{2}+a^{*2}+b^{*2}}$$

(4) Lactic Acid, Muscle Glycogen and pH

According to the instructions of the reagent kit produced by Beijing Solaibao Technology Co., Ltd., lactate and muscle glycogen were measured. The pH of fresh fish muscle was measured from the supernatant after homogenizing 2 g tissue with 20 mL distilled water and centrifuging at 5000× *g* rpm for 10 min at 4 °C.

(5) ATP and Its Metabolites

Based on the method described by Wang [63], high-performance liquid chromatography (HPLC) was used to determine the contents of ATP and its metabolites. with some modifications. 

Sample Preparation and Extraction:

Approximately 3 g of dorsal muscle from crucian carp, previously thawed at 4 °C, was minced under ice-bath conditions. The minced tissue was homogenized with 15 mL of 5% perchloric acid (PCA) and centrifuged at 5000× *g* r/min for 10 min at 4 °C. The supernatant was collected, and the residue was re-extracted with an additional 10 mL of 5% PCA, followed by centrifugation under the same conditions. The two supernatants were combined, and the pH was adjusted to 6.5 using 1 mol/L and 6 mol/L NaOH. The mixture was allowed to stand at 4 °C and then brought to a final volume of 50 mL with ultrapure water. A 2 mL aliquot of the solution was filtered through a 0.22 μm membrane and transferred into a vial for HPLC analysis.

HPLC Analysis:

Chromatographic separation was performed using a Shimadzu Shim-pack GIST-3 μm C18 column (2.1 mm × 100 mm) maintained at 30 °C. The mobile phase consisted of solvent A (15 mmol/L KH_2_PO_4_ and 15 mmol/L K_2_HPO_4_, *v*/*v* = 1:1, pH 5.55) and solvent B (methanol), with a flow rate of 1 mL/min and an injection volume of 10 μL. Detection was carried out at 254 nm over a total run time of 35 min using a gradient elution as follows: 0–8 min, 100% A; 8–10 min, B increased linearly to 3%; 10–15 min, B increased to 6%; 15–23 min, B increased to 15%; 23–28 min, B increased to 30%; 28–30 min, A returned to 100%.

The freshness index (K value) was calculated as the percentage of HxR and Hx in the total ATP-related compounds. The formula used to calculate the K value (%) is as follows:

$$K=\frac{HxR+Hx}{ATP+ADP+AMP+IMP+HxR+Hx}\times 100\%$$

### 3.13. Determination of caspase 3, caspase 8, caspase 9, bcl-2, bax in Fish Muscle

Using fluorescence quantitative PCR method, total RNA extraction, reverse transcription, and quantitative PCR were performed sequentially. The specific methods were based on Peng et al. [4], and the primer sequences are listed in Table 4.

### 3.14. Statistical Analysis

Each measurement was performed at least three times, and data are presented as the mean ± standard deviation based on the average of repeated measurements. Data were analyzed using SPSS 27.0 (IBM Corp., Armonk, NY, USA). One-way analysis of variance (ANOVA) was conducted, with polynomial linear contrasts applied for trend analysis. Post hoc multiple comparisons were performed using the least significant difference (LSD) and Duncan’s tests. A significance level of 0.05 was used for all analyses. Graphs were generated using GraphPad Prism 9 (GraphPad Software, San Diego, CA, USA).

## 4. Conclusions

Prolonged transportation negatively impacts fish survival, physiology, and muscle quality. Ginger extract added to water effectively alleviated stress (COR), and oxidative damage (SOD and GPX) and improved muscle quality during (shear force, drip loss) early transport but lost efficacy over time. In contrast, microencapsulated ginger extract provided sustained antioxidant protection, stabilized water quality, and improved survival and muscle quality. While the combination of NaCl and ginger extract accelerated water quality deterioration, the combination of eugenol and ginger extract effectively reduced ammonia accumulation, oxidative stress, and muscle damage. The observed effects may be attributed to the anesthetic properties of eugenol, which helped mitigate oxidative stress and enhance fish survival. Additionally, the combination improved muscle quality by increasing shear force, reducing drip loss, and inhibiting the extrinsic apoptotic pathway. Overall, the sustained-release formulation and eugenol–ginger combination showed strong potential to enhance fish welfare and product quality during transportation.

## Figures and Tables

**Table 1:** Changes in the color of fish muscle during transportation.

Transportation Time/h	Group	L*	a*	b*	*W*
0	CK	48.48 ± 1.37 ^Ba^	3.33 ± 0.88 ^Aa^	1.26 ± 0.61 ^Aa^	48.35 ± 1.44 ^Ba^
	GG	48.57 ± 1.23 ^Ba^	2.95 ± 0.26 ^Aa^	1.13 ± 0.34 ^Ba^	48.47 ± 1.24 ^Ba^
	MG	48.08 ± 1.27 ^Ba^	3.01 ± 1.36 ^Aa^	1.6 ± 0.48 ^Aa^	47.95 ± 1.34 ^Ba^
	NG	49.45 ± 1.15 ^Ba^	2.59 ± 0.66 ^Aa^	1.25 ± 0.58 ^Aa^	49.36 ± 1.16 ^Ba^
	EG	48.1 ± 0.78 ^Ba^	2.69 ± 0.77 ^Aa^	1.23 ± 0.39 ^Ba^	48.01 ± 0.79 ^Ba^
24	CK	46.93 ± 0.66 ^Bc^	1.96 ± 0.46 ^Bb^	1.68 ± 0.27 ^Aa^	46.86 ± 0.66 ^Bc^
	GG	47.64 ± 0.78 ^Bc^	2.79 ± 0.53 ^Aa^	2.25 ± 0.63 ^Aa^	47.51 ± 0.79 ^Bc^
	MG	50.52 ± 0.67 ^Aa^	2.27 ± 0.32 ^Aab^	1.89 ± 0.41 ^Aa^	50.43 ± 0.66 ^Aa^
	NG	51.31 ± 0.49 ^Aa^	2.21 ± 0.69 ^Aab^	2.03 ± 0.54 ^Aa^	51.21 ± 0.47 ^Aa^
	EG	49.34 ± 0.78 ^Bb^	2.86 ± 0.48 ^Aa^	2.03 ± 0.18 ^Aa^	49.22 ± 0.8 ^Bb^
48	CK	50.94 ± 0.75 ^Abc^	2.92 ± 0.48 ^ABa^	1.58 ± 0.47 ^Aa^	50.82 ± 0.73 ^Ab^
	GG	51.4 ± 0.51 ^Aabc^	1.94 ± 0.53 ^Bb^	1.68 ± 0.44 ^ABa^	51.32 ± 0.51 ^Aab^
	MG	50.71 ± 0.67 ^Ac^	1.92 ± 0.45 ^Ab^	1.52 ± 0.33 ^Aa^	50.65 ± 0.68 ^Ab^
	NG	51.97 ± 0.5 ^Aab^	1.67 ± 0.38 ^Ab^	1.33 ± 0.44 ^Aa^	51.92 ± 0.49 ^Aa^
	EG	52.37 ± 0.88 ^Aa^	1.59 ± 0.13 ^Bb^	1.38 ± 0.36 ^Ba^	52.32 ± 0.89 ^Aa^

Notes: Different uppercase letters indicate significant differences within the group at different time points (*p* < 0.05), while different lowercase letters indicate significant differences between groups at the same time point (*p* < 0.05). CK: Control group; GG: Ginger group; MG: Microcapsule group; NG: NaCl compound group; EG: Eugenol compound group.

**Table 2:** Changes in ATP and its metabolites of fish during transportation.

Transportation Time/h	Group	ATP (mg/100 g)	ADP (mg/100 g)	IMP (mg/100 g)	Hx (mg/100 g)	AMP (mg/100 g)	HxR (mg/100 g)	K (%)
0	CK	3.51 ± 0.19 ^Ba^	1.09 ± 0.05 ^Aa^	183.37 ± 2.18 ^Ca^	2.9 ± 0.17 ^Ba^	5.52 ± 0.08 ^Ba^	3.97 ± 0.19 ^Ca^	3.43 ± 0.02 ^Ba^
	GG	3.57 ± 0.16 ^Ba^	1.03 ± 0.07 ^Aa^	183.99 ± 1.59 ^Ba^	2.88 ± 0.15 ^Ba^	5.55 ± 0.07 ^Aa^	3.94 ± 0.24 ^Aa^	3.4 ± 0.04 ^Aa^
	MG	3.45 ± 0.1 ^Ba^	1.06 ± 0.09 ^Aa^	183.57 ± 2.13 ^Aa^	2.99 ± 0.16 ^Aa^	5.5 ± 0.05 ^Aa^	3.84 ± 0.24 ^Ba^	3.41 ± 0.05 ^Aa^
	NG	3.56 ± 0.17 ^Ba^	1.04 ± 0.09 ^Aa^	182.57 ± 0.94 ^Ca^	2.98 ± 0.17 ^Ca^	5.55 ± 0.07 ^Aa^	3.82 ± 0.21 ^Ba^	3.41 ± 0.05 ^Ba^
	EG	3.52 ± 0.16 ^Ca^	1.05 ± 0.08 ^Aa^	183.38 ± 1.78 ^Ba^	2.94 ± 0.16 ^Ba^	5.53 ± 0.07 ^Aa^	3.89 ± 0.22 ^Ba^	3.41 ± 0.04 ^Aa^
24	CK	5.57 ± 1.01 ^Aab^	0.64 ± 0.1 ^Ba^	202.9 ± 11.32 ^Ba^	3.52 ± 0.11 ^Aa^	6.5 ± 0.3 ^Aa^	4.66 ± 0.13 ^Ba^	3.66 ± 0.17 ^ABa^
	GG	4.63 ± 0.46 ^Ab^	0.62 ± 0.18 ^Ba^	200.66 ± 3.34 ^Aa^	3.46 ± 0.18 ^ABa^	4.46 ± 1.23 ^ABb^	4.18 ± 0.14 ^Aa^	3.51 ± 0.04 ^Aa^
	MG	5.25 ± 0.94 ^Ab^	0.31 ± 0.03 ^Bb^	219.03 ± 38.61 ^Aa^	3.53 ± 0.25 ^Aa^	3.37 ± 0.99 ^Bb^	4.11 ± 0.77 ^ABa^	3.31 ± 0.58 ^Aa^
	NG	6.55 ± 0.12 ^Aa^	0.34 ± 0.02 ^Bb^	206.19 ± 3.21 ^Ba^	3.88 ± 0.17 ^Ba^	3.82 ± 0.09 ^Bb^	3.44 ± 0.06 ^Bb^	3.27 ± 0.1 ^Ba^
	EG	6.56 ± 0.26 ^Aa^	0.29 ± 0.07 ^Bb^	198.33 ± 3.74 ^Ba^	2.21 ± 0.5 ^Bb^	3.66 ± 0.18 ^Bb^	3.43 ± 0.07 ^Cb^	2.63 ± 0.24 ^Bb^
48	CK	3.27 ± 0.41 ^Bb^	0.55 ± 0.07 ^Ba^	233.29 ± 14.72 ^Aa^	4.57 ± 0.68 ^Aa^	2.82 ± 0.18 ^Cb^	5.1 ± 0.23 ^Aa^	3.87 ± 0.23 ^Aa^
	GG	4.14 ± 0.86 ^ABb^	0.28 ± 0.04 ^Cb^	207.44 ± 8.26 ^Ab^	4.06 ± 0.76 ^Aa^	3.42 ± 0.89 ^Bb^	4.19 ± 0.11 ^Ac^	3.7 ± 0.46 ^Aa^
	MG	4.02 ± 0.91 ^Bb^	0.35 ± 0.07 ^Bb^	208.79 ± 6.14 ^Ab^	4.29 ± 1.51 ^Aa^	2.93 ± 0.83 ^Bb^	4.77 ± 0.36 ^Aab^	4.03 ± 0.7 ^Aa^
	NG	3.41 ± 0.36 ^Bb^	0.34 ± 0.07 ^Bb^	222.03 ± 11.6 ^Aab^	4.39 ± 0.36 ^Aa^	2.42 ± 0.16 ^Cb^	4.32 ± 0.48 ^Abc^	3.67 ± 0.17 ^Aa^
	EG	5.58 ± 0.29 ^Ba^	0.36 ± 0.05 ^Bb^	217.3 ± 18.3 ^Aab^	4.33 ± 0.67 ^Aa^	6.13 ± 1.71 ^Aa^	4.75 ± 0.3 ^Aab^	3.83 ± 0.44 ^Aa^

Notes: Different uppercase letters indicate significant differences within the group at different time points (*p* < 0.05), while different lowercase letters indicate significant differences between groups at the same time point (*p* < 0.05). CK: Control group; GG: Ginger group; MG: Microcapsule group; NG: NaCl compound group; EG: Eugenol compound group.

**Table 3:** Primers used for quantitative PCR.

Target Gene	Primer Sequence (5′→3′)	GenBank Accession Numbers	PCR Product Lengths
Heat shock protein 70 (*hsp70*)	S: ACACGACTGGTCCATTTCTGC	XM_026208615.1	71
	A: ACTTCGTTGTCTCTCCGGTC		
Heat shock protein 90 (*hsp90*)	S: ATGAGGACAAGGACAAACCGAA	KT985291.1	138
	A: GTCTTGTAGAAGGCTTTGTATCGTC		
Cysteine-aspartic acid protease 3 (*caspase 3*)	S: ACAGGCATGAACCAACGGAA	XM_026203855.1	174
	A: ACACACTAACGAAGCACAACG		
Interleukin 6 (*il-6*)	S: GACGGCTGTCTGTCCAGAAACT	XM_051873835.1	132
	A: GATGTCGTTGACCAGGGTTGAG		
Glyceraldehyde-3-phosphate dehydrogenase (*gapdh*)	S: AGGCATTCTGGGATACACGGAG	XM_026284269.1	242
	A: GATGGGAGAACGGTGGGTCA		

Note: “S” at the end of the primer name refers to the forward primer, and “A” refers to the reverse primer.

**Table 4:** Primers used for quantitative PCR.

**Target Gene**	**Primer Sequence (5′→3′)**	**GenBank** **Accession Numbers**	**PCR** **Product Lengths**
Cysteine-aspartic acid protease 3 (*caspase 3*)	S: ACAGGCATGAACCAACGGAA	XM_026203855.1	174
	A: ACACACTAACGAAGCACAACG		
Cysteine-aspartic acid protease 8 (*caspase 8*)	S: TACGACTGAACGAGCAAGCA	XM_026259219.1	141
	A: ATGCGTCACGTTGTAGCAGA		
Cysteine-aspartic acid protease 9 (*caspase 9*)	S: AACAAGACGTGACCAAGCCAG	XM_026241892.1	173
	A: GCGAAGGCTGTATGGGGACA		
B-cell lymphoma 2 (*bcl-2*)	S: CTGATGCCTTTTTGGCCGTTG	BAG06937.2	182
	A: CGACACTAGGCTCTTGCGA		
Bcl-2-associated X protein (*bax*)	S: CTTTGCGTGTCGGCTTGTC	XM_026262398.1	133
	A: CTCCCATCCACCCTGTTCC		
Glyceralde-hyde-3-phosphate dehydrogenase (*gapdh*)	S: AGGCATTCTGGGATACACGGAG	XM_026284269.1	242
	A: GATGGGAGAACGGTGGGTCA		

## Data Availability

Data will be made available on request.

## Supplementary Materials

The following supporting information can be downloaded at: https://www.mdpi.com/article/10.3390/ijms26167689/s1.
