# Supplementary material for: Mitigating Effect of Ginger Extract on Survival Rate and Muscle Quality of Crucian Carp (Carassius auratus) Under Transportation Stress
Source: Int J Mol Sci. 2025 Aug 8;26(16):7689. doi: 10.3390/ijms26167689 (PMC12386352; doi:10.3390/ijms26167689)
Supplement: Supplementary file 1 [file ijms-26-07689-s001.zip › ijms-3745873-supplementary.pdf]

Figure S1. Image of the microcapsule

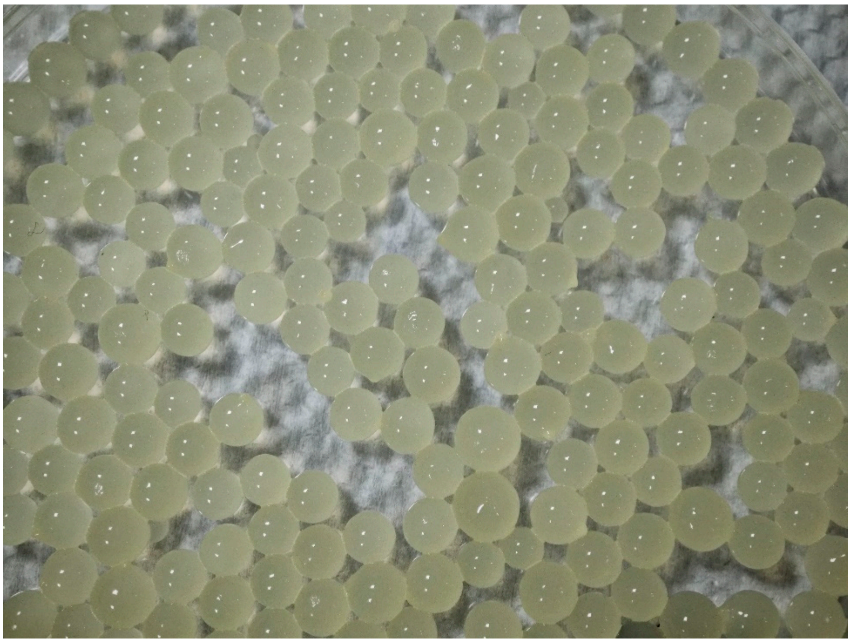

Table S1. Changes in the muscle fiber area and intermuscular distance of fish muscle during transportation.

Group	Muscle fiber area (μm^2)			Intermuscular distance (μm)		
	0 h	24 h	48 h	0 h	24 h	48 h
CK	7882.16 ±4556.22Aa	4669.9 ±4323.53Aa	4600.67 ±3441.05Aa	136.06 ±48.82Aa	103.73 ±51.09Aa	95.5 ±38.09Aa
GG	8339.67 ±2616.71Aa	5072.84 ±2858.16Ba	4126.85 ±1531.81Ba	122.43 ±14.36Aa	100.47 ±30.56ABa	92.5 ±22.03Ba
MG	7910.05 ±3741.64Aa	5929.95 ±3097.16ABa	4316.11 ±1829.08Ba	138.14 ±43.19Aa	108.46 ±27.24ABa	98.33 ±14.25Ba
NG	7939.33 ±2757.52Aa	4769.4 ±2194.4Ba	3904.91 ±2274.55Ba	142.74 ±33.26Aa	98.28 ±22.69Ba	91.69 ±29.81Ba
EG	7910.05 ±3741.64Aa	4893.89 ±1292.14Ba	4254.86 ±1324.21Ba	138.14 ±43.19Aa	113.51 ±9.88ABa	102.68 ±15.8Ba

Note: Different uppercase letters indicate significant differences within the group at different time points ($p < 0.05$), while different lowercase letters indicate significant differences between groups at the same time point ($p < 0.05$). CK: Control group, GG: Ginger group, MG: Microcapsule group, NG: NaCl compound group, EG: Eugenol compound group.
